# Peritoneal flap hernioplasty for repair of incisional hernias after orthotopic liver transplantation

**DOI:** 10.1007/s10029-021-02409-5

**Published:** 2021-04-22

**Authors:** M. F. Nielsen, A. de Beaux, B. Stutchfield, J. Kung, S. J. Wigmore, B. Tulloh

**Affiliations:** 1 Department of Upper GI Surgery, Royal Infirmary of Edinburgh, 51 Little France Cres, Edinburgh, EH16 4SA Scotland, UK; 2 Department of Surgery, Hospital of Southern Denmark, Aabenraa, Danmark

**Keywords:** Incisional hernia repair, Orthotopic liver transplantation, Peritoneal flap hernioplasty, Component separation, Short and long-term complications

## Abstract

**Background:**

Repair of incisional hernias following orthotopic liver transplantation (OLT) is a surgical challenge due to concurrent midline and transverse abdominal wall defects in the context of lifelong immunosuppression. The peritoneal flap hernioplasty addresses this problem by using flaps of the hernial sac to bridge the fascial gap and isolate the mesh from both the intraperitoneal contents and the subcutaneous space, exploiting the retro-rectus space medially and the avascular plane between the internal and external oblique muscles laterally. We report our short and long-term results of 26 consecutive liver transplant cases with incisional hernias undergoing repair with the peritoneal flap technique.

**Methods:**

Post-OLT patients undergoing elective peritoneal flap hernioplasty for incisional hernias from Jan 1, 2010–Nov 1, 2017 were identified from the Lothian Surgical Audit system (LSA), a prospectively-maintained computer database of all surgical procedures in the Edinburgh region of south-east Scotland. Patient demographics and clinical data were obtained from the hospital case-notes. Follow-up data were obtained in Feb 2020.

**Results:**

A total of 517 liver transplantations were performed during the inclusion period. Twenty-six of these (18 males, 69%) developed an incisional hernia and underwent a peritoneal flap repair. Median mesh size (Optilene Elastic, 48 g/m^2^, BBraun) was 900 cm^2^ (range 225–1500 cm^2^). The median time to repair following OLT was 33 months (range 12–70 months). Median follow-up was 54 months (range 24–115 months) and median postoperative stay was 5 days (range 3–11 days). Altogether, three patients (12%) presented with postoperative complications: 1 with hematoma (4%) and two with chronic pain (8%). No episodes of infection or symptomatic seroma were recorded. No recurrence was recorded within the follow-up period.

**Conclusion:**

Repair of incisional hernias in patients following liver transplantation with the Peritoneal Flap Hernioplasty is a safe procedure associated with few complications and a very low recurrence rate. We propose this technique for the reconstruction of incisional hernias following liver transplantation.

## Introduction

Incisional hernias are reported in 5–20% of cases following liver transplantation [1–3]. They are associated with a variety of risk factors including the male gender, the pathophysiology of the underlying liver disease, the nutritional status, obesity, ascites, wound complications, and the immunosuppressive treatment [4–7]. The repair of incisional hernias after liver transplantation presents a major surgical challenge owing to the complex nature of the incision and the quality of the tissue available for repair. The incision transects the muscles of the lateral abdominal wall, i.e. the internal and external oblique muscles, and the transversus abdominis muscle, and frequently also the rectus muscles [8]. Since this incision involves several muscle groups, it commonly results in complex hernias that may be difficult to repair. The repair is further complicated by the immunosuppressive treatment administered to prevent rejection. These drugs may interfere with tissue healing and increase the risk of postoperative complications [5, 9].

The risk of recurrence has improved over recent years, owing to the use of prosthetic mesh and tension-free repairs. While smaller hernias can successfully be repaired using a laparoscopic approach [10], larger, more complex hernias may necessitate open surgery to ensure a safe and effective repair.

The peritoneal flap technique was developed to facilitate the repair of large, complex hernias using the hernial sac to bridge the fascial gap irrespective of its location or orientation, providing the basis for a low-tension repair [11, 12]. The technique positions the mesh in the plane between the abdominal wall muscles and is useful for repair of both midline and lateral defects, which contrasts to component separation, which applies mainly to midline defects.

The present study aims to describe the peritoneal flap technique for the repair of incisional hernias after liver transplantation and to present the short and long-term results of this technique in these patients. The data demonstrates that the peritoneal flap technique is a safe procedure that can be used for the repair of incisional hernias in high-risk liver transplant recipients with few complications and a very low recurrence rate.

### Surgical technique

We have previously demonstrated that the peritoneal flap technique is a versatile approach that enables the repair of complex hernias [11, 13]. Due to the complex nature of the incision, the peritoneal flap approach with its ability to bridge both transverse [13] and midline [11] abdominal wall defects constitute an attractive alternative to component separation in liver transplant patients. The procedure involves a retro-rectus dissection medially and a dissection between the external and internal oblique muscles laterally. Alternatively, the extra-peritoneal plane accessed via a TAR approach can be used for the dissection within the lateral abdominal wall [14–17]. The description below aims to outline the technical aspects of the repair of complex incisional hernias in liver transplant recipients. The procedure is essentially a hybrid approach for repair of midline and transverse incisional hernias [11, 13].

**Fig. 1:**
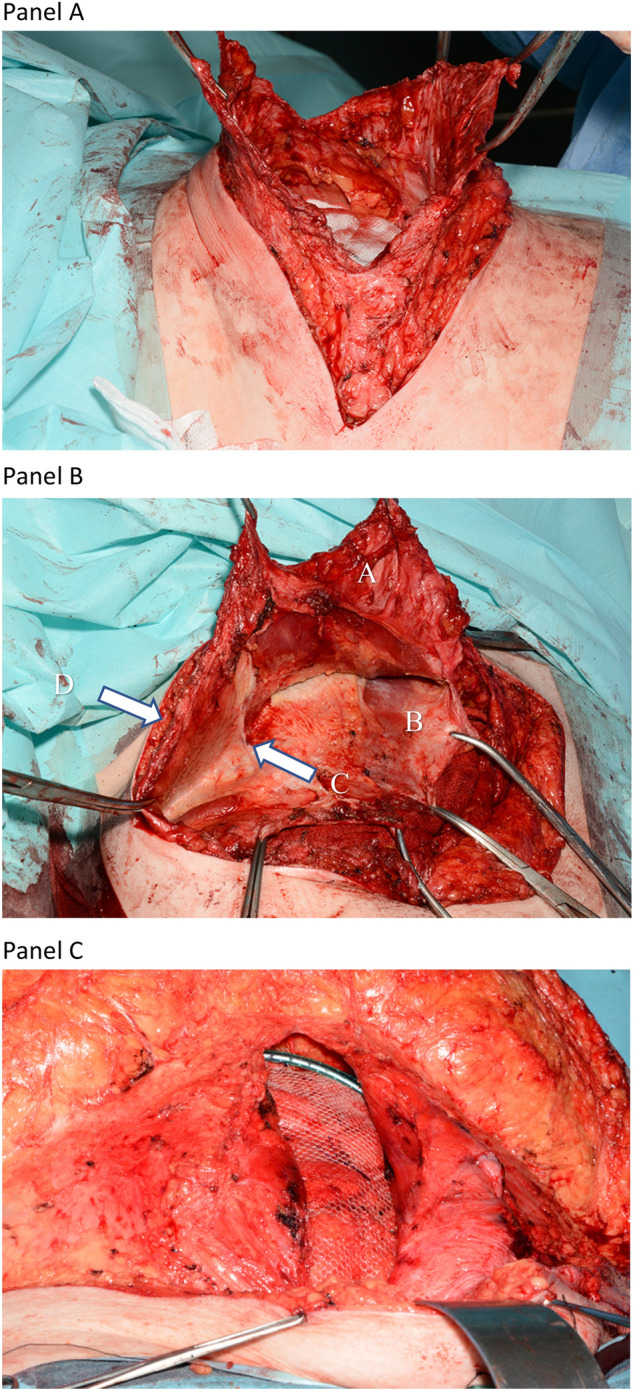
Peritoneal flap repair for a posttransplant incisional hernia. The skin is incised and the old scar removed. The hernia sac is incised over the full length of the defect (Panel A). The plane between the external and internal oblique muscles laterally, and deep to the rectus muscles medially is developed to create a space for the mesh (Panel B). **a** Preserved half of the hernial sac attached to the anterior fascial layer. **b** Posterior rectus sheath. **c** Linea semilunaris. **d** External oblique muscle. The external fascia is closed using a part of the hernial sac to bridge the fascial defect and obtain a non-tension repair. Note the drain placed in the retro-muscular plane (Panel C)

The scar is excised along with any redundant skin and scar tissue. Skin flaps are elevated to expose the sac and the defect’s margins over the entire length of the scar at the level of the deep fascia. The hernia sac is opened centrally over the full length of the defect in the line of the incision (Fig. 1, Panel A). Note that for a reverse-L-shaped incision, the opening of the sac is also reverse-L-shaped. Peritoneal adhesions within the sac and adjacent to the defect margins are divided.

In general, the anterior peritoneal flap is more conveniently left attached to the superior part of the transverse incision and the right anterior fascia of the midline component of the incision, and thus, the posterior flap is left attached to the posterior layers inferiorly (Fig. 1, Panel B). Medially, the retro-rectus plane is developed, as for a standard Rives-Stoppa-type repair [18, 19]. In both a reverse L-shaped incision and a subcostal incision, the retro-rectus space is entered on both sides. Laterally, the avascular plane between the external and internal oblique muscles is developed. The lateral and medial spaces are developed individually and then joined together across the linea semilunaris, which presents as a condensation of fascial layers at the edge of the rectus sheath (Fig. 1, Panel B).

The next stage is to join the avascular planes on the caudal and cephalad sides of the defect around the medial and lateral ends of the wound to create a common space for the mesh. Laterally this lies immediately deep to the EO aponeurosis and medially it is the retro-rectus space. Both spaces can be developed widely to accommodate a large mesh. This plane is the ideal place to position the mesh, because it enables an adequate overlap of the repair and takes into account the blood supply to the lateral and ventral parts of the abdominal wall [20, 21].

Closure of the abdominal cavity is performed by first suturing the deeper peritoneal flap to the cut edge of the opposite posterior musculo-fascial layers, which comprise the TA/IO muscle laterally and the posterior rectus sheath medially. The underlying peritoneal membrane is included in the repair. Once this is completed, the peritoneal cavity is isolated from the plane where the mesh is placed. The hernial defect is ‘bridged’ by the peritoneal flap without undue tension creating a common plane extending across the now-closed defect into the avascular spaces all around. A suitable mesh is cut to size and laid into this space with a generous overlap in all directions beyond the closed peritoneal suture line. The mesh should be trimmed as required to lie flat with no folding or curling. It is then fixed to the posterior musculo-fascial layer with interrupted sutures or tissue glue as an alternative. The repair is finally completed by closing the anterior layers of fascia/flap over the mesh, with the mesh as the “sandwich” filling.

Wide dissection within the tissue planes of the abdominal wall is important to obtain a large common space above and below the wound for mesh placement. A 5 cm overlap over the closed peritoneal suture line is recommended. Once the anterior fascial layer is closed over the mesh, the skin incision may be closed in a routine fashion. Subcutaneous drains are recommended to reduce the risk of seroma. Drains to the plane of the mesh may be used to prevent postoperative hematoma within the muscular compartment (Fig. 1, Panel C).

### Study methods

The present study is an uncontrolled case series from a single institution. Patients undergoing emergency or elective liver transplantation at the Scottish Liver Transplant Unit, Royal Infirmary of Edinburgh and subsequent incisional hernia repair from Jan 1, 2010, until Nov 1, 2017, were identified from the Lothian Surgical Audit (LSA) system, a prospectively maintained database of all surgical procedures in the Edinburgh region of south-east Scotland. Patients undergoing subsequent incisional hernia repair were identified and those in whom we used the peritoneal flap technique were selected for study. Emergency hernia repair cases were excluded. The LSA database also records all hospital attendances across our region, including any for postoperative complications, and is, therefore, a reliable tool to identify and register the incidence of recurrence as well as short and long-term complications.

Patient characteristics including age and sex of the patients, body mass index (BMI), operative details including defect size and mesh size, length of postoperative stay, and post-operative progress at 3 months were recorded from each patient’s case-notes (Table 1). Technical data concerning the results from preoperative abdominal CT, length of hospital stay, mesh size, and time of follow-up was likewise retrieved from patient files and recorded (Table 3). Postoperatively, patients were regularly followed up in the liver transplant clinic. Those with symptoms or signs of complications related to the abdominal wall repair were referred on for assessment by a hernia specialist. Recurrence was defined as “a protrusion of contents of the abdominal cavity through a defect in the abdominal wall at the site of the previous repair” [22, 23]. Imaging (CT or ultrasound) was performed to document the presence or absence of short and long-term complications and to confirm or refute the presence of recurrence.

**Table 1:** Patient characteristics

Characteristics	Value^a^	Range/percent
Number of patients included	26	
Gender distribution (male vs female)	18 vs 8	69.2 vs 30.8%
Age (years)	59.2 ± 6.6	43–69
Height (m)	1.69 ± 0.09	1.54–1.82
Weight (kg)	85.1 ± 13.6	62–105
BMI (kg/m^2^)	29.9 ± 4.1	22.5–37.6
ASA score	2.9 ± 0.3	2–3
Smoking	2	8%
Type 2 diabetes	5	19%
NAFLD/ALD	14	53.8%
HCV	3	11.5%
AIH	3	11.5%
PBC	2	7.7%
Acute liver failure	2	7.7%
Cryptogenic cirrhosis	1	3.8%
Carolis disease	1	3.8%

^a^Mean ± SD

## Results

Five hundred and twenty-nine liver transplant procedures were performed from Jan 1, 2010, to Nov 1, 2017. Patient’s notes were reviewed in Feb 2020. Postoperatively, 26 patients developed an incisional hernia within the review period (5%). All of these were repaired using the peritoneal flap method. Patient characteristics are depicted in Table 1. Liver transplantation was in these subjects performed for NAFLD/ALD (54%), HCV cirrhosis (12%), PBC (8%), acute liver failure (8%), cryptogenic cirrhosis (4%), and Carolis Syndrome (4%) (Table 2). Eight (31%) of patients were females and 18 (69%) males. The size of the hernial defect was recorded in 49% of cases assessed from preoperative CT scans. Postoperative CT scans were performed in 3 of the 26 study subjects (12%) for reasons unrelated to the hernia repair. All postoperative scans demonstrated an intact abdominal wall with no signs of recurrence, hematoma or persistent seroma. The incisional hernia repair was performed on average 34 months (range 12–70 months) after liver transplantation. Details of the hernias and the repairs are shown in Table 3.

**Table 2:** Immunosuppressive treatment

Diagnosis	Number of patients	Percent (%)
Prograf	1	4
Prograf + MMF	10	38
Prograf + MMF + Prednisolone	1	4
Prograf + azathioprine	6	23
Neoral + MMF	2	8
Not recorded	6	23

**Table 3:** Operative details

Characteristics	Value^a^	Range
Preoperative CT scan	48.6%	
Postoperative CT scan	12%	
Mesh size (cm^2^)	861 ± 313	225–1500
Length of hospital stay (days)	5.8 ± 2.0	3–11
Defect diameter on CT (cm)	8.6 ± 2.4	4–12.7
Postoperative follow-up (months)	54 ± 27	20–111
Abdominoplasty	46%	

^a^Mean ± SD

Early complications were recorded in 3 cases (12%) (Table 4). Patients were routinely reviewed in the hernia clinic at 3 months and thereafter in the liver transplant clinic. Follow-up ranged from 20 to 111 months with a median of 54 months. One patient developed a wound hematoma requiring surgery (4%) and two patients (8%) reported pain or discomfort 3 months after the repair. No cases of superficial wound infection or symptomatic seroma requiring surgical intervention were found, nor any cases of deep mesh infection. No episodes of skin necrosis were recorded.

**Table 4:** Outcome and complications

Problem	Numbner	Percent (%)
Skin necrosis	0	0
Superficial wound infection	0	0
Symptomatic seroma requiring reoperation	0	0
Wound haematoma reguiring reoperation	1	3.8
Chronic pain	2	7.7
Recurrence	0	0

No patients died within the follow-up period. Among the 26 incisional hernia repairs, none developed hernia recurrence during the follow-up period.

## Discussion

The repair of incisional hernias following liver transplantation constitutes a major surgical challenge [14, 24–27]. Approximately 30% of the abdominal wall repairs in our institution are performed for transverse hernias, 19% of these in liver transplant recipients [13]. While the repair of midline hernias commonly is performed using the Rives-Stoppa-Wantz procedure [18, 19], the repair of transverse defects is considerably more complicated, because the surgical procedure has to take into account not only the location and the size of the hernial defect, but also the complex anatomy of the abdominal wall [14, 21, 25, 27].

Liver transplantation is most commonly performed through a transverse or oblique subcostal incision, which extends through the rectus muscles into the left lateral abdominal wall. Since several muscle groups are involved, complex hernias may evolve that may be challenging to repair. Furthermore, the repair of these hernias are performed in the context of immunosuppressive treatment which, when administered in combination with sirolimus and steroids, impairs wound healing [5, 9]. They are therefore prone not only to develop incisional hernias after the transplant, but are also more susceptible to recurrence following the incisional hernia repair [1].

The peritoneal flap hernioplasty was initially developed as a modification of the da Silva triple-layer repair first described in 1979 [28]. The method uses reflected layers from the abdominal wall as well as excess tissue derived from the hernial sac to traverse the fascial gap thereby reconstructing the fascial defect while increasing abdominal domain and preserving the integrity of the muscular layers in the abdominal wall. We have described this repair for midline incisional hernias [11, 12], and more recently also for the repair of transverse incisional hernias [13]. This technique is highly applicable to hernias arising through transverse incisions as long as the complex anatomy of the abdominal wall away from the midline is taken into account. Applying this repair to a reverse L-shaped incision is also no more than an exercise in applied anatomy, combining the midline and transverse elements of the peritoneal flap repair simultaneously.

The present study demonstrates that the peritoneal flap technique can provide a robust and lasting repair in liver transplant patients with complex incisional hernias. The reason for this outcome is severalfold. First, the method creates a large pocket within the abdominal wall that enables a generous overlap of the mesh [20]. Second, the reflected hernial sac is used as a form of fascial “extension” to bridge the defect and create a low-tension repair. Larger hernias with larger sacs produce larger flaps, so the technique can be applied to a variety of hernia sizes. By using autologous tissue derived from the hernial sac to “bridge” the fascial defect in a low-tension manner, the abdominal domain is increased at the site of the repair. This is in contrast to component separation techniques, which increase domain laterally. Third, by using a large piece of mesh between the peritoneal flaps and securing it to the strong fascial layers beyond the margins of the defect, the peritoneal flap technique creates a tri-laminar complex comprising sac-mesh-sac, which provides strength to the repair but also the opportunity for ingrowth of tissue into the mesh. Indeed, results from an animal study provide evidence that suggests that peritoneal stem cells derived from the hernial sac may be able to form aponeurotic tissue [29]. This implies that the peritoneal flaps may have the ability to transform into strong fascia. If so, that may at least in part explain the low recurrence rate observed in the present study.

Despite the complexity of the repair, no cases of superficial or deep wound infection were recorded and only one case (4%) developed a hematoma requiring surgical intervention. Although this low complication rate may have occurred simply by chance, we doubt this to be the case, since the data is consistent with what we previously have reported in non-transplant patients [11, 13]. Nonetheless, we cannot rule out that there may have been cases with mild episodes of cellulitis that has subsided on antibiotics alone. These patients are commonly treated in the transplant clinic, and unless an abscess has developed, these small, superficial infections may not have been reported and therefore not included in this review.

It should be emphasized that the mean size of the fascial defect reported in Table 3 was determined from preoperative CT scans, which in the present study was performed in 49% of cases. We conduct pre-operative CT scans selectively to aid in choosing the appropriate strategy of the repair. Such decisions can usually be based on clinical examination alone, but in cases with potential loss of domain and/or in cases where the abdominal wall is difficult to assess, a preoperative CT scan may provide helpful information regarding the quality of muscle and fascia tissue available for the repair.

The Intra-Peritoneal Onlay Mesh (IPOM) procedure has been suggested as an alternative to open incisional hernia repair in liver transplant patients [30, 31]. This procedure carries a low risk of infection and seroma and is therefore an attractive approach in high-risk transplant recipients. However, current expert opinion is that intraperitoneal mesh should be avoided when possible owing to the risk of intestinal adhesions, erosion, and fistulation [32, 33]. While a laparoscopic or robotic approach is feasible for smaller hernias, it remains to be determined whether larger defects also can be adequately repaired with these techniques owing to the lack of support centrally for the bridging mesh repair, and the high risk of post-operative bulging [34]. Bulging is uncommon after the peritoneal flap hernioplasty [11], presumably because of a combination of the triple-layer, wide mesh overlap, and strong tissue ingrowth into the mesh.

Two recent papers have addressed the challenge of open incisional hernia repair in transplant patients [35, 36]. In these studies, the incidence of short and long-term outcomes in transplant recipients undergoing incisional hernia repair with component separation (CST) was reported. A recurrence rate of 5.7% from 35 cases was observed by Zolper et al. [36] after a median follow-up of three years, using a variety of CST techniques and mesh locations. The most common use of mesh was in the intraperitoneal sublay position, but some intermuscular sublay repairs were also performed. Because of this heterogeneity it is difficult to attribute the low recurrence rate to any particular technique. Tastaldi et al. [35] reported a recurrence rate of 25% after a median follow-up of 13 months using a transversus abdominis release (TAR). The discrepancy in recurrence rate between the latter and the present study, with no recurrences after a median of 54 months follow-up, is striking. While this difference may be related to subject characteristics, and perhaps to the assiduousness of postoperative follow-up, it may also reflect the difference in surgical technique. The plane of dissection with the peritoneal flap technique is centered around the previous incision line irrespective of its orientation, while the TAR repair uses a midline skin incision to access the retro-rectus space, then continues laterally through the transversus abdominis layer into the extraperitoneal plane on of the lateral abdominal wall one or both sides. It appears that this mode of dissection opens up a significantly larger plane in the abdominal wall than is required for the peritoneal flap repair, which may potentially explain the difference in the short and long-term recurrence and complication rates in the two studies. A randomized study would be helpful to further explore the difference in outcome between the peritoneal flap and the TAR repair.

In conclusion, we report in the present paper the results from 26 liver transplant patients undergoing the peritoneal flap repair for incisional hernia. These results demonstrate that this is a reliable method with few complications associated with low recurrence and complication rates in high-risk patients with complex incisional hernias.

## Abbreviations

Abdominoplasty: Removal of excessive skin and fat
BMI: Body mass index
EO: External oblique muscle
IO: Internal oblique muscle
IPOM: Intra-peritoneal onlay mesh
LSA: Lothian surgical audit system
OLT: Orthotopic liver transplantation
TA: Transversus abdominis muscle
TAR: Transversus abdominis release
